# Suppression of wingless-type MMTV integration site family, member 1 expression by small interfering RNA inhibits U251 glioma cell growth *in vitro*

**DOI:** 10.3892/ol.2014.2647

**Published:** 2014-10-30

**Authors:** LUN DONG, XIAO-CHUN DUAN, CHONG-XU HAN, HENGZHU ZHANG, YONGKANG WU

**Affiliations:** 1 Department of Neurosurgery, Clinical Medical College of Yangzhou University, Yangzhou, Jiangsu 225001, P.R. China; 2 Central Laboratory, Clinical Medical College of Yangzhou University, Yangzhou, Jiangsu 225001, P.R. China

**Keywords:** wingless-type MMTV integration site family, member 1, RNA interference, glioma, plasmid, U251 cells, proliferation

## Abstract

A Wingless-type MMTV integration site family, member 1 (*Wnt-1*) RNA interference expression vector was constructed during the present study, which was used to transfect the glioma U251 cell line and investigate its effect on glioma. Two 21-base oligonucleotides complementary to the coding sequence that was flanking the loop sequence were designed to form a DNA hairpin template for the target small interfering RNA (siRNA). The siRNA templates were cloned into the siRNA expression vector, pGPU6/green fluorescent protein (GFP)/Neo and the sequence was confirmed by DNA sequencing. The pGPU6/GFP/Neo-short hairpin RNA (shRNA)-*Wnt-1* vector was subsequently transfected into U251 cells, and reverse transcription polymerase chain reaction and western blot analysis were used to evaluate the *Wnt-1* gene silencing effect on U251 cell growth by MTT assay and flow cytometry. The Wnt-1 protein expression was significantly reduced following transfection with the recombinant plasmid, as determined by western blot analysis of the transfected U251 cells. This transfection exhibited a significantly higher death rate, as shown by MTT. Thus, the present study demonstrated that the pGPU6/GFP/Neo-shRNA-*Wnt-1* vector inhibited Wnt-1 protein expression. However, further investigations regarding the *Wnt* signaling pathway in glioma pathogenesis are required.

## Introduction

Wingless-type MMTV integration site, member 1 (*Wnt-1*), the first identified member of the *Int-1* family, was named by Nusse in 1991 (1). The first *Int-1* was termed *Wnt-1*, as *Int-1* and the *Wnt* gene family were homologous. The *Wnt-1* gene consists of 805 adenines, 1,523 cytosines, 1,320 guanines and 874 thymines. Current studies have proposed that the Wnt-1 protein controls cell growth, proliferation, secretion of key signaling molecules, mediates information flow between cells and stem cells, and is important in neural development. *Wnt-1* gene expression has been found to closely correlate with the development of numerous types of tumor (1–7). You *et al* (8), as well as other studies, have also indicated that *Wnt-1*-target genes may be potential cancer therapy targets (9,10). Preliminary experiments in the present study found that the Wnt-1 protein expression in human glioma was significantly higher than in normal brain tissue, and is closely associated with the degree of malignancy. Therefore, the aim of the current study was to further investigate the role of *Wnt-1* in glioma by RNA interference (RNAi) targeting.

## Materials and methods

### Materials

U251 glioma cells were purchased from the Cell Bank of the Shanghai Institute of Biochemistry and Cell Biology (Shanghai, China). The vectors used were as follows: pGPU6/green fluorescent protein (GFP)/Neo-short hairpin RNA (shRNA)-GAPDH served as a positive control and pGPU6/GFP/Neo-shNC served as a negative control. The pGPU6/GFP/Neo expression plasmid was purchased from Wuhan Genesil Biotechnology Co., Ltd (Wuhan, China). Transfections were performed using liposome Lipofectamine^TM^ 2000 reagent and TRIzol that were purchased from Life Technologies (Foster City, CA, USA). The *Wnt-1* primary antibody was obtained from Neomarkers Inc. (Fremont, CA, USA), while the horseradish peroxidase-conjugated monoclonal goat anti-rabbit secondary antibody was purchased from ZhongShan Bio-Tech Co., Ltd. (Guangzhou, China). High glucose Dulbecco’s Modified Eagle’s Medium (DMEM) was purchased from Life Technologies and fetal calf serum was purchased from Hangzhou Sijiqing Biological Engineering Materials Co., Ltd. (Hangzhou, China). The restriction enzymes (*Bam*HI, *Pst*I and *Bbs*I), DNA markers, agarose gel DNA purification kit version 2.0, T4 DNA ligase Taq DNA polymerase and polymerase chain reaction (PCR) primers were purchased from Takara Bio, Inc. (Shiga, Japan). Little mention plasmid kit, and propidium iodide (PI) and MTT reagents were purchased from Beyotime Institute of Biotechnology (Nantong, China). M-MLV reverse transcriptase was purchased from Promega Corporation (Madison, WI, USA).

### Small interfering RNA (siRNA) target sequence design

The full-length *Wnt-1* gene sequence was retrieved from GenBank (NM_005430) and the siRNA design principles were obtained from Takara Biotechnology special siRNA design software [Takara Biotechnology (Dalian) Co., Ltd., Dalian, China) to select a 21-nt siRNA target template. The shRNA selected TTCAAGAGA loop structure was maintained to avoid formation of a termination signal and the shRNA transcription termination sequence was composed of a T6 structure. CACC was added to the sense strand of the template to form a *Bbs*I complementary sticky end and antisense strand template. GATC was added to the 5′ end and was digested with *Bam*HI to generate complementary sticky ends. When the first siRNA base was not a G, then an additional CACC was added after the G. The following primers were used: Sense, 5′-ACGGCGTTTATC TTCGCTATC-3′ and antisense, 3′-TGCCGCAAATAGAAG CGATAG-5′ for *Wnt-1*. The following hairpin single-stranded oligonucleotide sequence was then designed and synthesized: Sense, 5′-CACCGACGG CGTTTATCTTCGCTATCTTCAAGAGAGATAGCGAAG ATAAACGCCGTTTTTTTG-3′ and antisense, 5′-GATCCA AAAAAACGGCGTTTATCTTCGCTATCTCTCTTGAAG ATAGCGAAGATAAACGCCGTC-3′ for *Wnt-1*. The oligonucleotides were synthesized by the Shanghai Jima Company (Shanghai, China).

### Construction of Wnt-1 targeting pGPU6/GFP/Neo-Wnt-1 vector for RNAi

Synthetic single-stranded oligonucleotides were dissolved in Tris-EDTA buffer (pH 8.0; 10mM Tris-HCl, 1 mM EDTA), diluted to 100 μmol/l and annealed to form an shRNA template (5 μl justice chain, 5 μl antisense strand, 5 μl annealing buffer and 35 μl double distilled (dd) H_2_O to a 50-μl total volume). The shRNA template was annealed on a PCR instrument (ABI7900HT, Invitrogen Applied Biosystems, Foster City, CA, USA) according to the following procedure: 95°C for 5 min, 85°C for 5 min, 75°C for 5 min and 70°C for 5 min. The template was stored at 4°C to generate a 10-μM concentration of shRNA. The template solution was diluted 500-fold to a final concentration of 20 nM for ligation. Restriction digestions were performed with 2μg pGPU6/GFP/Neo, according to the following conditions: 5 μl 10X buffer G (Shanghai PureOne Biotechnology Co., Shanghai, China), 2 μl *Bbs*I enzyme, 2 μl *Bam*HI enzyme, 2 μg pGPU6/GFP/Neo and ddH_2_O to a total volume of 50 μl. Following digestion at 37°C for 1 h, the product concentrations were examined by agarose gel electrophoresis, using the agarose gel DNA purification kit version 2.0 and diluted to 50 ng/μl. Ligations were performed in 2 μl 10X T4 ligation buffer, 1 μl pGPU6/GFP/Neo (*Bbs*I + *Bam*HI), 1 μl shRNA template, 0.5 μl T4 DNA ligase (5 Weiss U/μl) and 30 μl ddH_2_O to a total volume of 20 μl at room temperature for 2 h.

### Plasmid identification

Competent *Escherichia coli* DH5α cells were grown on lysis buffer medium plates containing kanamycin (Beyotime Institute of Biotechnology) at 37°C for 18 h. Six colonies were selected and used to inoculate the kanamycin-containing LB liquid medium (50 μg/ml), which was agitated using a blender (Beijing Tianshi Tianli Medical Device Technology Development Center, Beijing, China) overnight. The plasmids were extracted by alkaline lysis and digested sequentially with *Bam*HI and *Pst*I. The positive recombinant vectors were digested with *Bam*HI, but not *Pst*I. Two clones were selected for each vector and sequenced by Invitrogen Applied Biosystems (Shanghai, China).

### Cell culture

The U251 cell lines were grown at 37°C in a 5% CO_2_ humidified atmosphere with high glucose DMEM medium (containing 10% fetal bovine serum (FBS), 100 U/l penicillin, 100 mg/l streptomycin and 10% fetal calf serum).

### Plasmid transfection

Transfected cells (3×10^5^) were grown to 70–80% confluence for two days, and subsequently inoculated in six-well plates (35 mm), with 2 ml 10% FBS (Chongqing Manuik Technology Co., Ltd., Chongqing, China) per well. Prior to transfection, the liposome/DNA was incubated for 10 min, and replaced with 1 ml fresh serum-free 10% FBS (Chongqing Manuik Technology Co., Ltd.). Based on the pretest results, the cells were cultured with Lipofectamine^TM^ 2000 reagent (Life Technologies) and plasmid at a ratio of 6 μl:2 μg, respectively, according to the manufacturer’s instructions, and incubated at 37°C in a 5% CO_2_ humidified atmosphere. After 5 h, 2–3 ml fresh growth medium was added. The cells were collected the next day and fresh 10% fetal calf serum was added. The cells were then cultured for 48 h to observe any GFP expression. The cells were divided into the following four transfection groups: pGPU6/GFP/Neo-*Wnt-1*, pGPU6/GFP/Neo-shRNA-GAPDH (positive control), pGPU6/GFP/Neo-shNC (negative control) and a phosphate-buffered saline (PBS) control. GFP expression was used to evaluate the transfection efficiency 48 h following transfection and was measured by 488-nm excitation via fluorescence microscopy (Olympus BX51; Olympus America Inc., Center Valley, PA, USA).

### Reverse transcription (RT)-PCR detection of Wnt-1 mRNA expression level

The primer design software Premier 5.0 (Premier Biosoft, Palo Alto, CA, USA) was used to design the following primers: Sense, 5′-CTGCCTCTCTTCTTCCCCTT-3′ and antisense, 5′-TCACAGCTGTTCAATGGCTC-3′ for *Wnt-1* (251-bp product); and sense, 5′-CCATGT TCGTCATGGGTGTGAACCA-3′ and antisense, 5′-GCCAGT AGAGGCAGGGATGATGTTC-3′ for the internal reference, GAPDH (246-bp product). Preamplification of GAPDH was used to assess the efficacy of RT, with a semi-quantitative PCR reaction run in parallel, which served as an internal reference. TRIzol was used to extract the total cellular RNA and first-strand cDNA synthesis was performed according to the manufacturer’s instructions. The total RNA (2 μg) was used for RT, in addition, 0.1 μg oligo-dt, denatured at 65°C for 10 min and placed immediately in an ice bath for 5 min, was added to 5 μl 5X PCR buffer [Takara Biotechnology (Dalian) Co., Ltd.], 1 μl dNTP (10 mM each), 100 units RNAsin, 10 units M-MLV reverse transcriptase and water, which was added to produce a total volume of 25 μl. The samples were incubated at 42°C for 60 min following a 5-min 95°C denaturalization step, subsequently placed in an ice bath for 3 min and stored at −20°C to conserve the RNA template for the PCR. The RNA template was then converted into cDNA for use in the RT-PCR, which was performed in a total reaction volume of 50 μl, containing 5 μl 10X buffer, 4 μl MgCl_2_ (25 mM), 1 μl dNTP (10 mM each), 0.5 μl primer (50 pmol/μl), 0.5 μl downstream primer (50 pmol/μl), 0.3 μl Taq enzyme (5 U/μl), 2 μl cDNA template and 30–50 μl paraffin oil. PCR reactions were performed as follows: 95°C denaturation for 5 min; 30 cycles of 94°C for 30 sec, 50°C annealing for 30 sec and 72°C for 1 min; with a final step at 72°C for 5 min. The products were subjected to 2% agarose electrophoresis and ethidium bromide staining. Images were captured using the IBM 586 computer-controlled Gel Doc 1000 imaging system (Bio-Rad, Hercules, CA, USA).

### Western blot analysis

Western blot analysis was used to detect Wnt-1 protein expression prior to and following U251 transfection. Total cellular protein was determined by SDS-PAGE gel electrophoresis. The proteins were transferred to nitrocellulose membranes (Whatman Inc., London, UK) for protein immunoassays and the primary monoclonal mouse anti-human proto-onocogene Wnt-1 antibody (Boster Biotechnology Co., Ltd., Wuhan, China) was used at a dilution of 1:100, with the secondary monoclonal rabbit anti-mouse antibody (Boster Biotechnology Co., Ltd.) at 1:500.

### MTT cell proliferation assay

U251-transfected cells were subjected to trypsin digestion following 24 h. Single cell suspensions were prepared in culture medium containing 10% fetal calf serum, transferred into 96-well plates at a final volume of 200 μl and grown at 37°C in a 5% CO_2_ humidified atmosphere for 48 h. Each MTT well contained a 20-μl MTT solution (5 mg/ml), which was incubated for 4 h. At culture termination, the medium was carefully aspirated and the supernatant discarded. Each well contained 150 μl dimethyl sulfoxide and was agitated for 10 min to fully dissolve the crystals. ELISA was used to record the absorbance at 490 nm and the cellular proliferation rate was calculated as follows: Cellular proliferation rate (%) = (experimental group/absorbance value of blank control group) × 100.

### Flow cytometry (FCM) detection of cell cycle

Trypsinized cells were transfected with plasmids 48 h following digestion. The cell suspension was then centrifuged in a clean centrifuge tube using a high speed centrifuge (Beijing Era Beili Centrifuge Co., Ltd., Beijing, China) at 507 xg for 5 min. The culture medium was discarded and washed three times with cold PBS. The cells were resuspended by adding 75% alcohol, fixed for 30 min in ethanol and centrifuged at 1,917 xg for 5 min, followed by washing three times with PBS. The cells were then resuspended in 100 μl PBS and 2.5 μl RNase (10 mg/ml), followed by 25 μl PI pyridine (10 mg/ml) and staining for 30 min. The cells were examined using a FACSAria flow cytometer (BD Biosciences, Franklin Lakes, NJ, USA) to determine the G_1_/G_0_, G_2_/M and S phase fractions.

### Statistical analysis

SPSS 10.0 (SPSS Inc., Chicago, IL, USA) was used for analysis of variance. Data are presented as the mean ± standard deviation. The means between two groups were compared using Student’s t-test, while multiple comparisons were analyzed using the F-test. P<0.05 was considered to indicate a statistically significant difference.

## Results

### Plasmid verification

**Figure 1:**
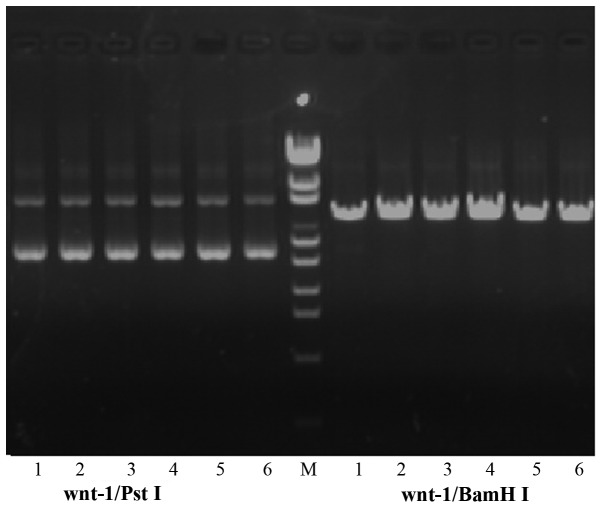
Restriction map of the Wnt-1 plasmid.

Successful digestion by *Bam*HI, but not *Pst*I confirmed construction of the recombinant plasmid vector (Fig. 1). Furthermore, sequencing verified that the targeted *Wnt-1* gene had been inserted into the pGPU6/GFP/Neo complete shRNA vector.

### RT-PCR detection of Wnt-1 gene mRNA expression level changes following RNAi

**Figure 2:**
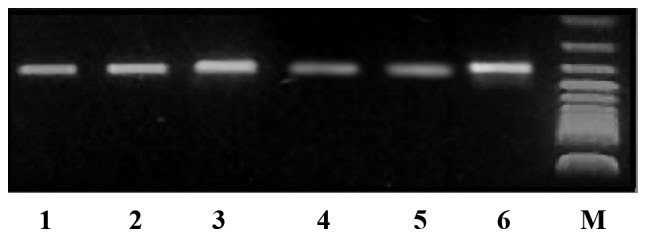
Reverse transcription polymerase chain reaction detection of the *Wnt-1* gene mRNA expression levels following plasmid transfection. Lanes: 1, Blank control; 2, negative control; 3, positive control; 4 and 5, transfected pGPU6/GFP/Neo-shRNA-*Wnt-1*; 6, GAPDH internal reference; and M, 50-bp DNA marker.

Transfected cells were characterized by semi-quantitative RT-PCR 48 h following transfection to detect the pGPU6/GFP/Neo-shRNA-*Wnt-1*-positive cells. The *Wnt-1* mRNA content had decreased by 63% compared with the controls, while the negative and positive control groups did not exhibit any significant differences (Fig. 2). Compared with the controls, no significant differences in Wnt-1 gene mRNA expression levels were observed between the negative and positive control group (99 and 100%, respectively).

### Knockdown of Wnt-1 protein expression by plasmid transfection

**Figure 3:**
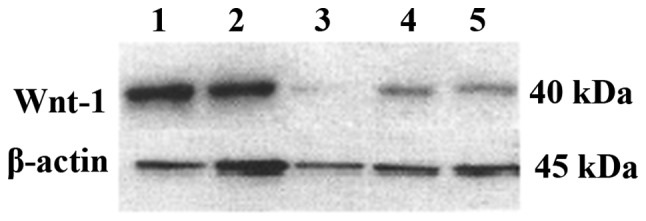
Western blot analysis of the transfected Wnt-1 plasmid. Lanes: 1, Blank controls; 2, negative controls; 3, transfected positive controls; and 4 and 5, pGPU6/GFP/Neo-shRNA-*Wnt-1* group.

Western blot analysis showed that the Wnt-1 protein expression was significantly reduced following transfection. Although the negative control did not change significantly, the expression of the positive control decreased. This may be the result of excessive cell death, which reduced the amount of extracted protein (Fig. 3).

### MTT cell proliferation assay

The results of the MTT assay indicated that the positive and negative controls exhibited decreased cell growth following pGPU6/GFP/Neo-shRNA-*Wnt-1* transfection. The positive controls that were transfected with pGPU6/GFP/Neo-shRNA-GAPDH showed decreased cell survival compared with the pGPU6/GFP/Neo-shRNA-*Wnt-1* transfected samples, however, survival at 48 h was not identified to be statistically significant.

### FCM test results

No statistically significant differences were identified between the positive and negative control groups at 48 h post-transfection (P>0.05), which indicated that the plasmid and transfection reagents were not toxic. The positive controls transfected with pGPU6/GFP/Neo-shRNA-*Wnt-1* were predominantly arrested at G_0_/G_1_ and the number of cells in the S phase was decreased (P<0.05). Furthermore, the number of replicating cells decreased, which indicated that U251 proliferation was inhibited (Table I).

## Discussion

The *Wnt* signaling pathways are important in cellular differentiation and proliferation in a variety of species, and are involved in the formation of certain types of tumor (11–13). The *Wnt*/β-catenin signaling pathway is considered to be a classical signaling pathway, which has been investigated in-depth (14). *Wnt* signal transduction mediates other processes, including *Wnt*/Ca^2+^ signaling, planar cell polarity, spindle orientation and asymmetric cell growth (11,14). *Wnt* binding of the Frizzled receptor inhibits the effect of glycogen synthase kinase 3-β, axin, and adenomatous polyposis coli protein complexes that are involved in β-catenin phosphorylation and degradation. Non-phosphorylated β-catenin is not degraded, however, accumulates in the cytoplasm, prior to translocation to the nucleus. β-catenin then binds to the lymphocyte enhancer factor/T cell-specific transcription factor, activating the target genes, *c-myc*, *cyclin D* and *MMP7*, leading to cellular proliferation and tumor formation. Therefore, certain studies have hypothesized that targeted therapy of *Wnt* signaling may inhibit tumor formation and growth (15).

Gliomas are the most common type of primary intracranial tumor and more effective therapeutic strategies are required to stop glioma progression. The *Wnt*/β-catenin signaling cascade is an important signal transduction pathway in human cancer (16). The human *Wnt* family consists of 19 members that regulate cell proliferation, differentiation, motility and fate during embryonic development and tumorigenesis. *Wnt* members bind to the cell surface receptors, Frizzled and low density lipoprotein receptor-related protein, and transduce their signals through β-catenin-dependent and -independent intracellular signaling pathways (17). *Wnt-1* was one of the first signaling molecules to be identified and it is considered to be the first step in the classical *Wnt*/β-catenin signaling pathway (18,19). Increasing evidence indicates that interplay between the *Wnt*/β-catenin and PI3K/AKT signaling cascades is involved in tumor development and progression. However, the mechanism of this in glioma is not well understood (20). Although a number of studies have identified a variety of tumors that exhibit abnormal *Wnt-1* expression (1,10), abnormal expression is rare in glioma. In our previous study, it was demonstrated that *Wnt-1* gene expression is increased in glioma, with little or weak expression identified in normal brain tissue. Furthermore, *Wnt-1* expression showed a positive correlation with the glioma grade (15). The present study indicated that targeting *Wnt-1* gene expression by RNAi knockdown inhibits the growth of human glioma cells. This demonstrates that glioma growth may be closely correlated with Wnt-1 protein expression. Malignant glioma growth may involve activation of the *Wnt-1* signaling pathway (21), however, the specific underlying mechanism requires further investigation (22).

Current RNAi methods are important for investigating gene function (18,23,24) and it is hypothesized that the *Wnt* signaling pathway may be ideal for targeted drug development. However, few studies have used *Wnt-1* gene RNAi. Certain studies have used fragments of chemically synthesized siRNA in transfected cells (25,26), or plasmids lacking a reporter gene (27), which complicates the assessment of transfection efficiency and the identification of stable cell lines. A previous study described the transfection of siRNA targeting *Wnt-1* into SH-SY5Y neuroblastoma cells using Lipofectamine^TM^ 2000 (28). *Wnt-1* and β-catenin protein expression decreased following transfection with siRNA, as did the cell number, and was accompanied by abundant floating dead cells. Additionally, the SH-SY5Y cells transfected with siRNA targeting *Wnt-1* showed less viability.

In conclusion, the current study used a pGPU6/GFP/Neo carrier plasmid containing a GFP reporter, which detects cellular expression efficiency and Kanamycin/G418 resistance, into stable cell lines. Therefore, siRNA targeting *Wnt-1* was used to investigate which region of the *Wnt-1* gene may be targeted as a novel hotspot (29). The results of the present study revealed that miR-106a-5p is a tumor suppressor gene in astrocytomas. The overexpression of miR-106a-5p inhibits astrocytoma cell proliferation, migration, and invasion and promotes apoptosis. In addition, FASTK was found to be a direct target for miR-106a-5p. Although much remains to be elucidated in terms of the role of miR-106a-5p in the pathogenesis of astrocytomas, miR-106a-5p presents a novel potential therapeutic target for the treatment of astrocytomas.

## Figures and Tables

**Table I:** Cell cycle changes of each group (mean ± standard deviation; n=4).

Group	G_0_/G_1_ phase (%)	S phase (%)	G_2_/M phase (%)
Transfection pGPU6/GFP/Neo-shRNA-Wnt-1	79.5±0.6	16.8±1.0	3.7±0.5
Positive control	78.5±0.4	11.6±0.6	10.0±0.6
Negative control	67.2±1.6	26.5±0.6	5.6±1.1
Blank control	67.4±0.7	27.2±0.4	5.1±0.5

The percentage of U251 cells in G_0_/G_1_ phase in the transfection and postive groups was higher than that of the negative control and blank groups (P<0.01). By contrast, the percentage of U251 cells in S phase in the transfection and postive groups was lower than that of the two control groups (P<0.01). No significant difference was identified between the transfection and postive groups in G_0_/G_1_ phase or S phase (P>0.05).
